# Activation of HER family members in gastric carcinoma cells mediates resistance to MET inhibition

**DOI:** 10.1186/1476-4598-9-121

**Published:** 2010-05-26

**Authors:** Simona Corso, Elena Ghiso, Virna Cepero, J Rafael Sierra, Cristina Migliore, Andrea Bertotti, Livio Trusolino, Paolo M Comoglio, Silvia Giordano

**Affiliations:** 1 Institute for Cancer Research and Treatment, University of Torino Medical School, Candiolo (Torino), Italy; 2 University Health Network, Ontario Cancer Institute and Princess Margaret Hospital, Toronto, ON, M5G 2M9, Canada

## Abstract

**Background:**

Gastric cancer is the second leading cause of cancer mortality in the world. The receptor tyrosine kinase MET is constitutively activated in many gastric cancers and its expression is strictly required for survival of some gastric cancer cells. Thus, MET is considered a good candidate for targeted therapeutic intervention in this type of tumor, and MET inhibitors recently entered clinical trials. One of the major problems of therapies targeting tyrosine kinases is that many tumors are not responsive to treatment or eventually develop resistance to the drugs. Perspective studies are thus mandatory to identify the molecular mechanisms that could cause resistance to these therapies.

**Results:**

Our *in vitro *and *in vivo *results demonstrate that, in MET-addicted gastric cancer cells, the activation of HER (Human Epidermal Receptor) family members induces resistance to MET silencing or inhibition by PHA-665752 (a selective kinase inhibitor). We provide molecular evidences highlighting the role of EGFR, HER3, and downstream signaling pathways common to MET and HER family in resistance to MET inhibitors. Moreover, we show that an *in vitro *generated gastric cancer cell line resistant to MET-inhibition displays overexpression of HER family members, whose activation contributes to maintenance of resistance.

**Conclusions:**

Our findings predict that gastric cancer tumors bearing constitutive activation of HER family members are poorly responsive to MET inhibition, even if this receptor is constitutively active. Moreover, the appearance of these alterations might also be responsible for the onset of resistance in initially responsive tumors.

## Background

Many efforts have been focused in better understanding the mechanisms of malignant transformation, resulting in the identification of molecules playing a crucial role in tumor growth. The race to discover compounds that specifically inhibit these targets is giving promising results, and many of these drugs successfully entered clinical trials, opening the era of the "targeted therapies" [1].

Cancer is a multigenic disease arising from the accumulation of different alterations of genes controlling cell proliferation and/or apoptosis [2]. However, recent studies in preclinical models demonstrated that tumor cells may be dependent on a single oncogene for their proliferation and survival. In fact, the specific inactivation of that oncogene leads to apoptosis of cancer cells and to tumor regression. This phenomenon, known as "oncogene addiction" [3], provides a further rationale for the use of targeted therapies. However, only a fraction of patients respond to these therapies, even if the molecular target of the drug is present in the cell. Moreover, almost invariably, responsive patients develop pharmacological resistance and undergo relapse, often due to the activation of alternative signaling pathways [4]. One of the major challenges of targeted therapies is, therefore, to know in advance which pathways could mediate resistance to the treatment and to find ways to circumvent these hurdles.

Gastric cancer is the second leading cause of mortality in the world and the first one in Asia. Despite the improvement of surgical techniques and the recent availability of new chemotherapic regimens, the outcome of patients with clinical advanced disease is usually poor. The identification of molecules altered in gastric cancers has led to the possibility of hitting them by use of specific targeted drugs. Among them is the receptor for Hepatocyte Growth Factor (HGF), encoded by the *MET *gene, that promotes a complex biological program called "invasive growth", inducing cells to break intercellular junctions, acquire a motile/invasive phenotype and escape apoptosis [5]. The improper activation of this program, due to MET deregulated activation, confers proliferative and invasive/metastatic ability to cancer cells [6]. Recent studies demonstrated that MET plays a role in a high percentage of human tumors [7]. In gastric cancers this receptor is frequently constitutively activated; activation is usually associated with receptor overexpression, that can be due to gene amplification. Moreover, MET activation can also result from infection of gastric cells by Helicobacter Pylori, a known predisposing factor for development of gastric cancer.

We and others have shown that gastric cancer cells bearing amplification of the *MET *gene and overexpression of the receptor, are "addicted" to this oncogene, since its inhibition results in impairment of tumor growth [8-10]. On these bases, MET is considered a good target in gastric cancer.

Recently, molecules targeting MET have gained access to clinical trials and results are expected soon [11]. Experience acquired from other RTKs has shown that only a percentage of patients respond to targeted therapies, even in the presence of the altered molecular target, and that almost invariably also responding patients develop resistance during treatment. Therefore, we were interested in identifying pathways whose activation could vicariate the signaling driven by MET. Several studies have shown the presence of a biochemical and functional interplay between MET and the HER (Human Epidermal Receptor) family of RTK (reviewed in [12,13]). This family of receptors is frequently altered in gastric cancers where they are constitutively activated, mainly as consequence of gene amplification. Moreover, in patients with advanced gastric cancer, co-expression of c-Met and HER2 has been associated with poorer survival compared to overexpression of either one [14].

In our work we show that in gastric cancer cell lines "addicted" to MET, activation of HER family members, through ligand stimulation or mutational activation, contributes to overcome MET inhibition. This is due to the partial overlap of downstream signaling pathways common to MET and HER family. Moreover, we provide evidence that resistance to MET inhibition generated in cell lines by treatment with high doses of PHA-665752 is largely due to HER members overexpression.

## Results

### Ligand-dependent activation of HER family members induces resistance to MET inhibition in gastric cancer cells

**Figure 1:**
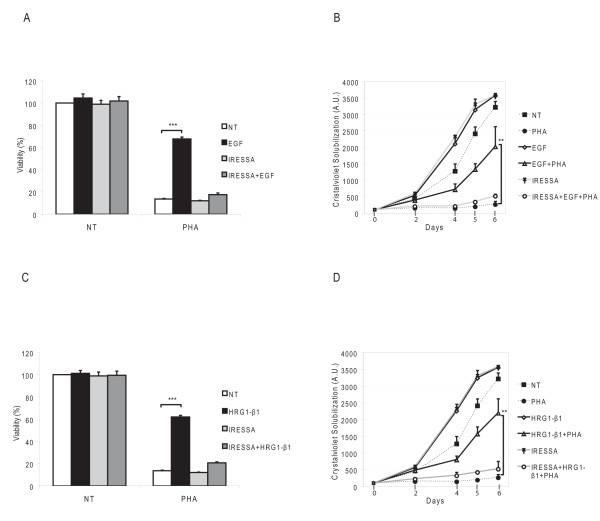
**Activation of HER family members rescues PHA-impaired cell viability and growth**. (A,C) Viability assays. GTL16 cells were untreated (NT) or treated with the PHA (250 nM), in the indicated conditions (EGF 50 ng/ml, HRG1-β1 10 ng/ml, or Gefitinib 250 nM). MET inhibition led to a strong decrease in cell viability compared to untreated cells (considered as 100%). Activation of HER members, upon EGF or HRG1-β1stimulation, conferred resistance to MET inhibition (*** P < 0,001). The specificity of the effect is shown by its loss in the presence of Gefitinib. (B,D) Growth curves. GTL16 cells were either untreated (NT) or treated with the indicated molecules. The block of cell growth induced by PHA was overcome by EGF or HRG1-β1 stimulation (** P < 0,01).

Cancer cell lines bearing *MET *gene amplification have been found to be "addicted" to MET []. GTL16 gastric cancer cells [15] are the prototype of "MET addicted cells", containing 11 copies of the *MET *locus [16], located on a marker chromosome [17]. The gene is actively transcribed and translated, leading to over-expression of the MET protein with a constitutive, ligand-independent, activation [18]. Indeed, when GTL16 cells were cultured in the presence of a well characterized and specific MET inhibitor, PHA-665752 (subsequently referred to as PHA) [19], their viability and growth ability were strongly impaired (Fig. 1A-1D).

There are several evidences of interplays between MET and HER family receptors [12,13]; moreover, signaling networks assembled by oncogenic EGFR and MET show significant overlapping [20]. We thus stimulated PHA-treated cells with ligands of the EGF family, to see if they could activate critical signaling pathways able to rescue cell viability. As shown in Fig. 1A, 1B, when Epidermal Growth Factor (EGF) was added to the culture medium, cells were able to significantly overcome the block of cell growth induced by PHA. A similar resistance to the effect of PHA could be induced also by Heregulin-β1 (HRG1-β1, Fig. 1C, 1D), known to bind HER3 and to induce its heterodimerization with the other family members [21]. To formally prove that the observed resistance depends on the activation of EGFR, upon formation of homodimers or heterodimers with other HER members, the same experiments were performed in the presence of Gefitinib, a specific EGFR inhibitor. As shown in Fig. 1A-1D, the ability of EGF and HRG1-β1 to stimulate cell viability and growth was lost in the presence of the inhibitor.

**Figure 2:**
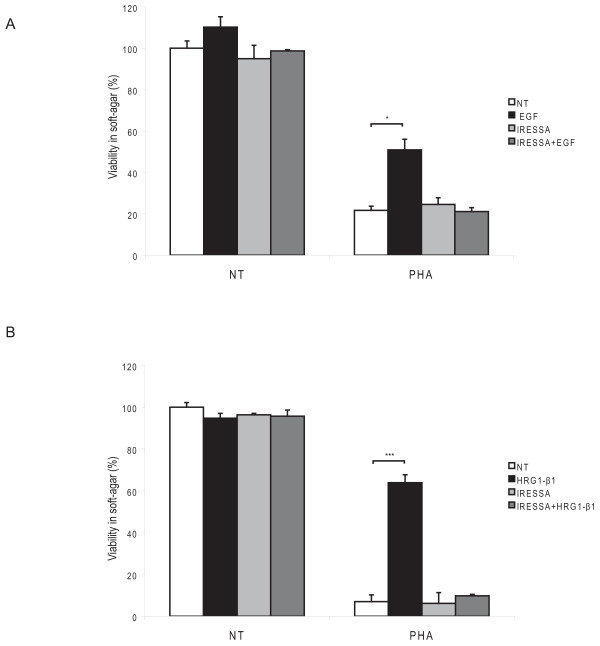
**Activation of HER family members rescues PHA-impaired anchorage-independent growth ability**. (A,B) GTL16 cells were grown in agar for 2 weeks and the amount of viable cells grown in colonies was evaluated with the Alamar Blue dye. MET inhibition with PHA (250 nM) resulted in impairment of cell ability to grow in an anchorage-independent manner compared to untreated cells, considered as 100%. Stimulation with EGF (50 ng/ml), or HRG1-β1 (10 ng/ml), rescued the ability of PHA-treated cells to form colonies (*P < 0,05; *** P < 0,001).

Functional assays evaluating cell growth in adherent conditions do not fully recapitulate the biological properties of tumor cells and, in particular, their ability to survive and grow in the absence of cell/substrate adhesion. Therefore, we performed soft-agar assays to evaluate if EGF and HRG1-β1 could induce resistance to MET inhibition also in conditions of anchorage-independent growth. As shown in Fig. 2A, 2B, while PHA-treated cells originated very few colonies in soft agar, the addition of either EGF or HRG1-β1 recovered their ability to grow in anchorage-independent manner. Also in this case, resistance to PHA induced by EGF and HRG1-β1 was abrogated by Gefitinib (Fig. 2A, 2B).

To verify if the observed behaviour was peculiar to GTL16 cells or if it was shared by other gastric cancer cells, bearing *MET *overexpression due to gene amplification (MKN45, SNU5, Hs746T gastric carcinoma cell lines), we treated them with PHA, in the absence or in the presence of either EGF or HRG1-β1. The expression of MET and of the members of the EGFR family in these cell lines is shown in the Additional file 1. Also in these cell lines, HRG1-β1 and/or EGF partially recovered cell ability to grow in the presence of PHA (Additional file 2), suggesting that HER family activation can interfere with MET targeting in gastric cancer cells (the poor response to HRG1-β1 observed in Hs746T cells is paralleled by the low expression of HER2/HER3 in this cell line (Additional file 1). The ability of HER family ligands to induce resistance to PHA in soft agar growth was also observed in MKN45 cells (the only other cell line able to grow in adhesion-independent conditions) (Additional file 2).

Altogether these findings suggest that the activation of the HER family receptors confers resistance to PHA-665752 in gastric cancer cells displaying MET overexpression due to gene amplification. Remarkably, the ability to overcome the effect of MET inhibition is not common to every growth factor, since neither MSP (Macrophage Stimulating Protein), nor IGF1 (Insulin like growth factor 1), for which GTL16 cell express the cognate receptors (data not shown), share this property with EGF family ligands (Additional file.3).

### MET trans-phosphorylation is not essential for the rescue by HER family members

**Figure 3:**
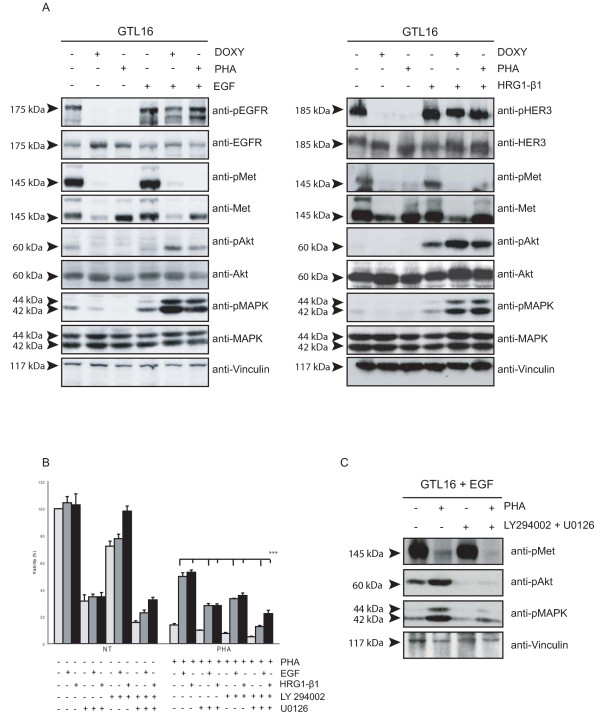
**Activation of AKT and MAPK pathways is required for resistance to MET blocking**. A) WB analysis of total GTL16 lysates. PHA treatment or MET silencing (DOXY) resulted in strong impairment of MET, EGFR, HER3, AKT and p42/44 MAPK phosphorylation. In these conditions, stimulation with EGF (5 ng/ml) or HRG1-β1 (10 ng/ml) restored AKT and MAPK activation. B) Cell viability assay in PHA-inhibited or not GTL16 cells. The presence of AKT and MAPK inhibitors abrogated the resistance to MET inactivation obtained with the two ligands (*** P < 0,001), while each inhibitor alone has only a partial effect.. C) WB analysis to control the effectiveness of LY294002 and U0126.

**Figure 4:**
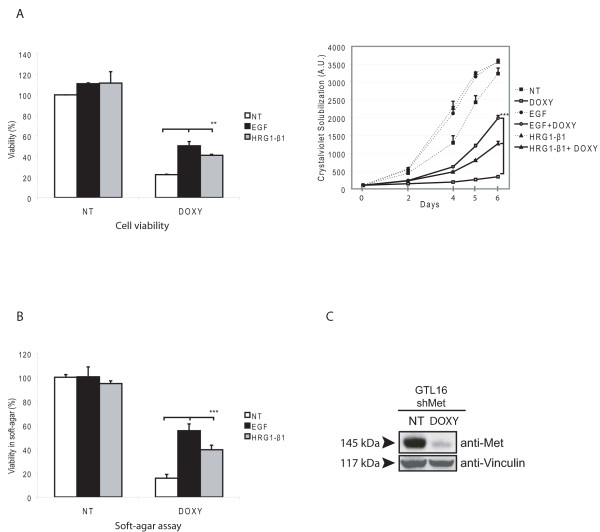
**MET trans-phosphorylation is not essential for the rescue exerted by HER family members**. A) Cell viability assay (*left*) and growth curve (*right*). GTL16 cells transduced with a doxycycline-inducible shRNA system were treated (DOXY) or not (NT) with doxycycline (1 μg/ml) to silence MET, and stimulated or not with EGF (50 ng/ml) or HRG1-β1 (10 ng/ml). Activation of HER members partially rescued inhibition of cell viability and growth induced by MET silencing (** P < 0,01). B) Cells were grown in agar and the number of viable cells was quantified with the Alamar Blue dye. Stimulation with EGF or HRG1-β1 partially rescued anchorage-independent growth ability in MET-silenced cells (DOXY; *** P < 0,001). (C) WB analysis showing effectiveness of MET silencing, 48 hours upon addition of doxycycline.

It is well documented in several experimental systems that MET and EGFR can interact and trans-phosphorylate each other (reviewed in [12]). This cross-talk also exists in GTL16 cells, where EGFR is basally tyrosine-phosphorylated, as consequence of MET constitutive activation; inhibition of MET kinase activity, in fact, results in EGFR dephosphorylation (Fig. 3). As tyrosine kinase inhibitors do not prevent RTK trans-activation due to other interacting receptors, we wondered whether the ability of EGFR to rescue MET inhibition could be due to trans-phosphorylation of the tyrosines located in the MET tail, acting as docking sites for most signal transducers [22]. To investigate this point, we took advantage of a RNA interference system able to silence MET in an inducible manner [10]. Upon doxycycline-induced MET silencing (Fig. 4C), GTL16 cells were strongly inhibited in their viability and in their anchorage-dependent and independent growth ability (Fig. 4A, 4B). However, in all the biological assays performed, the treatment with EGF or HRG1-β1 could overcome the effect of MET silencing (Fig. 4A, 4B), similarly to what seen with PHA. Since the silencing of MET was not complete, we cannot completely rule out the possibility that transphosphorylation may play a role in resistance. However, similar results obtained by chemical inhibition and by silencing suggest that the ability to overcome resistance is probably not due to MET trans-phosphorylation by EGFR, but, very likely, to the activation of MET-independent and parallel pathway(s).

To understand which biochemical events, downstream HER family, are responsible for the observed resistance to MET blocking, we analyzed the levels of AKT and MAPK activation in GTL16 cells (untreated, treated with PHA or silenced for MET expression), not stimulated or stimulated with EGF or HRG1-β1. As shown in Fig. 3A, a remarkable AKT and MAPK activation was observed after stimulation with EGF or HRG1-β1, upon MET inhibition or silencing. Notably, AKT activation was stronger when induced by HRG1-β1 compared to EGF stimulation. Phosphorylation of both AKT and MAPK was abrogated in the presence of Gefitinib, demonstrating its dependency on EGFR activation (data not shown).

To evaluate the role of the HER-dependent AKT and MAPK activation in conferring resistance to MET inhibition/silencing, we performed viability assays in the presence of specific AKT and MAPK inhibitors (LY294002 and U0126, respectively), whose activity was tested by Western blot (Fig. 3C). As shown, the presence of both inhibitors abrogated the ability of EGF and HRG1-β1 to overcome MET targeting (Fig. 3B), while each single inhibitor had only a partial effect. These data suggest that activation of AKT and MAPK pathways is required for resistance to MET blocking.

### Constitutive activation of HER family members prevent the *in vitro *and *in vivo *effectiveness of MET inhibition

**Figure 5:**
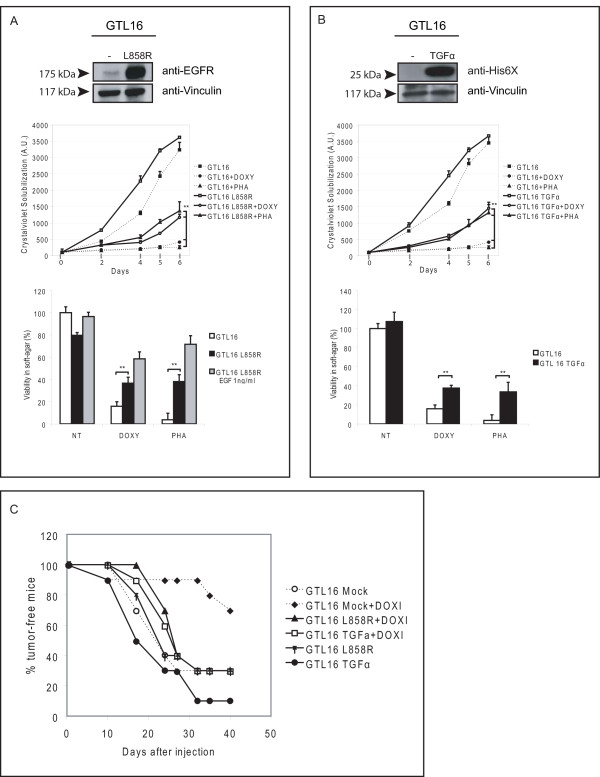
**Mutated EGFR or TGFα autocrine production impair the biological effects of MET targeting**. A-B) Cells, bearing the doxycycline-inducible MET shRNAs, were transduced with the mutated form of EGFR (L858R) (A) or with the Hys-tagged TGFα (B). *Top*, WB showing the expression of transduced molecules, compared to cells transduced with the empty vector; *Upper graph*, growth curves of GTL16, GTL16-L858R (A) or GTL16-TGFα (B). The effect of MET silencing (DOXY) or inhibition (PHA) was compared with untreated cells. After six days, only cells bearing EGFR-L858R or TGFα were able to grow when MET was inactivated (A ** P < 0,01; B *** P < 0,001). *Lower graph*, anchorage-independent growth assays. Cells having an activated EGFR (GTL16-L858R, A or GTL16-TGFα, B) formed colonies in soft-agar in the absence of MET signaling, while wt cells did not (A-B ** P < 0,01). Stimulation of GTL16-L858R cells with a physiological concentration of EGF (1 ng/ml) resulted in further increase of anchorage-independent growth, only in the absence of MET signaling. C) The cells used for biological assays were injected subcutaneously in CD1 nude mice. MET silencing was maintained *in vivo *by adding doxycycline (1 mg/ml) to mice drinking water. The graph shows the Kaplan-Meier-like analysis of tumor latency. After 40 days, 70% of mice injected with GTL16 cells and treated with doxycycline were tumor-free. On the contrary, only 30% of mice injected with GTL16-L858R and GTL16-TGFα were tumor-free, despite MET silencing. EGFR-L858R and TGFα had no significative effect in promoting tumor growth in untreated GTL16.

The most common EGFR activating alterations in human tumors are receptor point mutations (such as the L858R mutation, [23]) and the onset of TGFα autocrine production [24,25]. We thus investigated if the presence of these pathological alterations could induce resistance to MET inhibition in GTL16 cells. Through lentiviral transduction, we obtained GTL16 cells - already bearing the inducible shRNA system against MET [10] - stably expressing either the constitutively active EGFR-L858R (Fig. 5A, top panel) or TGFα (Fig. 5B, top panel). Cells transduced with an empty vector were generated as control. The transduced cells were tested for their ability to grow when MET was silenced or kinase-inhibited. As shown in Fig. 5A, cell expressing the EGFR-L858R mutant were able to partially overcome the effect of MET silencing/inhibition in all the assays. In cells growing in anchorage-independent conditions, the ability to induce resistance to MET blocking was further increased by the stimulation of mutant EGFR with physiological concentrations of EGF (a situation closely resembling a possible *in vivo *scenario). As expected, the effect of EGFR-L858R was abolished by Gefitinib (data not shown).

Similar results were obtained when GTL16 cells were transduced with the TGFα cDNA. As shown in Fig. 5B, also the autocrine-mediated activation of EGFR impaired PHA/shRNA effects on cell growth and colony formation. This suggests that constitutive activation of HER members, frequent in human tumors, can contribute to resistance to MET targeted therapies.

In order to verify the *in vivo *relevance of our findings, we performed xenograft experiments in mice. GTL16 cells expressing the inducible shRNA system to silence MET and then transduced either with an empty vector, or the EGFR-L858R mutant, or TGFα, were subcutaneously injected in nude mice. After one week, half of the mice of each experimental group (Mock, EGFR-L858R and TGFα) received doxycycline to silence MET in the tumor. As shown in Fig. 5C, MET silencing strongly delayed tumor onset in mice injected with control cells. In fact, after 40 days of MET silencing, the incidence of visible tumors was only 20%. However, tumors expressing EGFR-L858R or having the TGFα autocrine production were considerably resistant to MET silencing, as demonstrated by a complete rescue in tumor incidence (Fig. 5C). The expression of EGFR -L858R or TGFα does not significantly promote tumor growth in untreated cells (expressing MET). These data demonstrate that activating mutations of EGFR and TGFα autocrine loop can impair the effect of MET silencing *in vivo*.

### HER family members contribute to the onset of secondary resistance to MET inhibition

**Figure 6:**
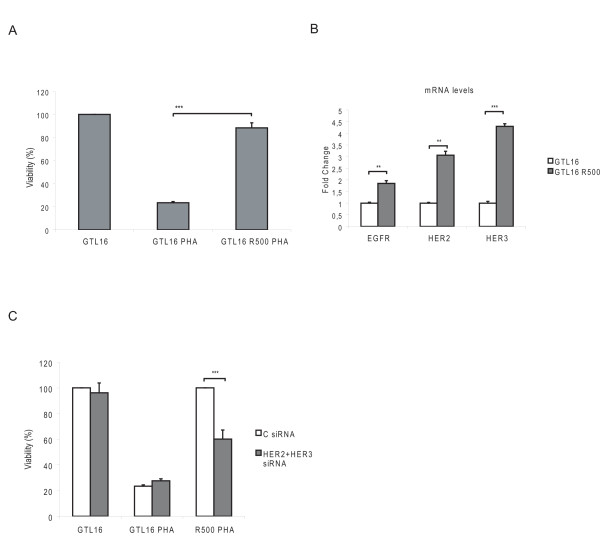
**HER family members contribute to onset of resistance to PHA treatment**. (A) Cell viability of GTL16 cells resistant to 500 nM PHA (GTL16 R500) compared to wt cells, grown in the presence of the drug for 96 hours. Growth of GTL16 R500 was not affected by the PHA. (B) Expression levels of HER family members in GTL16 cells wt or resistant to PHA, evaluated by Real time PCR. Absolute values were normalized to GAPDH. (C) Cell viability upon silencing of HER2/HER3. In GTL16 cells expression of HER2 and HER3 was silenced. While the silencing of the two receptors did not affect viability of wt cells, in GTL16 R500 the absence of HER2 and HER3 resulted in increased sensibility to PHA (*** P < 0,001).

To verify if HER members are involved in secondary resistance to MET inhibition, we continuously treated GTL16 cells with a dose of 500 nM PHA, mimicking a hypothetical clinical treatment regimen. After few months of PHA administration, cells developed resistance to the drug. In fact, while GTL16 parental cells treated with 500 nM PHA displayed an almost complete abrogation of growth, the resistant cells were only slightly affected by PHA (about 10% of cell viability reduction compared to untreated parental cells) (Fig. 6A). The analysis of these cells revealed that the *MET *gene was neither mutated nor amplified, and that other master regulators of cell proliferation, such as *H-RAS *and *K-RAS*, *B-Raf *and *PI3KCA *had none of the known mutations (data not shown). We then analyzed the HER family status, finding that the resistant cells showed a significant increase in the expression level of these receptors (especially HER2 and HER3), compared to parental cells (Fig. 6B). No mutations neither gene amplification were present in EGFR (exons 18, 19, 21, data not shown). In order to verify if the overexpression of HER2 and HER3 could be responsible, at least partially, for the development of resistance, we silenced both receptors in parental and in resistant cells (Additional file 4) and tested the viability of these cells in the absence or presence of PHA. Interestingly, we observed that HER2/HER3 silencing significantly reduced the ability of resistant cells to grow in the presence of PHA (Fig. 6C), with no significant effect on the parental counterpart.

Collectively, these findings demonstrate that alterations in HER family members can actually contribute to the onset of secondary resistance to PHA in initially responsive tumor cells.

## Discussion

The clinical experience derived from use of drugs targeting molecules that play critical roles in human tumors has shown that their efficacy critically depends on the presence of the altered target in the neoplasm [26]. However, even in these conditions, a response to the inhibitor is seen only in a fraction of patients (primary resistance) [27]. Moreover, even in responding patients in which the drugs are initially successful in impairing tumor growth, their efficacy decreases or is abrogated in a short time period, due to appearance of "secondary resistance" [4]. Most commonly, primary resistance is due either to constitutive activation of pathways downstream to the targeted molecule or to the engagement of alternative or redundant parallel signaling pathways that vicariate the lack of signal due to target inhibition. Secondary resistance can be due either to the same mechanisms, or to genetic alterations of the target, such as gene amplifications (rendering the amount of available drug not sufficient to block the target) or the appearance of point mutations (that prevent the interaction between the target and the drug). The recent availability of drugs that simultaneously inhibit multiple targets or the possibility to perform association therapies able to block synergistic signal transduction pathways has underlined the importance of identifying these functional and biochemical interactions, potentially involved in the appearance of resistance to targeted drugs.

Gastric cancer is an aggressive cancer, constituting a major cause of cancer-related deaths worldwide. Even if traditional therapies such as surgery, chemotherapy and radiotherapy have improved in recent years, patients with advanced disease have a poor prognosis, with a 5-year survival of less than 30%. For this reason, there is an absolute need for the integration in the treatment of this cancer of new drugs, targeting the genetic lesions present in the tumor. Molecular analyses performed in gastric cancer samples have shown that among the genes frequently altered in this tumor are tyrosine kinase receptors of the MET and HER families. The MET gene has been shown to be amplified in human gastric cancers and gastric cancer cell lines; amplification is known to be responsible for receptor overexpression and ligand-independent constitutive activation. Activating mutations have also been identified in some tumors of this histotype [6]. The role of the *MET *gene in human tumors has been firmly established [11] and it has also been demonstrated that genetic alterations of *MET *can be selected for the long-term persistence of the transformed phenotype as gene amplification is more frequent in metastatic lesions rather than in primary tumors [28]. Moreover, "in vitro" and preclinical models have shown that tumor gastric cells displaying *MET *gene amplification are "addicted" to the constitutive activity of this receptor for their growth and maintenance [8-10], thus suggesting that patients affected by this cancer could be ideal candidates for anti-MET targeted therapies. Indeed, clinical trials evaluating the effect of MET inhibition in these patients are ongoing [11]. It is also very puzzling to note that Helicobacter Pylori, a well known risk factor for this neoplasm, requires MET activation to exert its pro-tumorigenic effects [29]. Several reports have also identified in gastric cancers quantitative and qualitative alterations of members of the HER family, the most frequent being gene overexpression and amplification, even if also activating mutations have been detected [30,31]. Clinical trials targeting HER family members are thus ongoing in patients affected by gastric cancers [32]. It is important to note that in patients with advanced gastric cancer, co-expression of c-MET and HER2 has been associated with poorer survival compared to overexpression of either one[14]. These data thus suggest that, in some cases, co-targeting of both these molecules could be of clinical importance.

Several experimental evidences suggest the existence of biochemical and functional interplays between the members of the HER family and MET. Moreover, recent studies have shown that resistance to Gefitinib can be due to *MET *amplification [33]. In this case, MET overexpression and constitutive activation leads to HER3 trans-phosphorylation and activation of HER3-dependent survival pathways. In these cells, co-inhibition of MET and EGFR reverted resistance to Gefitinib. Since MET plays a role in mediating resistance to EGFR inhibition, we wondered if also the reversal was true. Some works have shown that, *in vitro*, activation of HER family members can lead to MET phosphorylation, but the role of this interplay has never been evaluated *in vivo *and in the contest of cells resistant to MET inhibitors [34,35].

As works conducted on other RTKs highlighted the ability of laboratory models to identify clinically relevant mechanisms of drug resistance, the aim of our work was to try to evaluate, *in vitro *and in preclinical models, the possible role of HER family receptors in mediating primary resistance to MET inhibition.

We took advantage of gastric MET-addicted tumor cell lines that stop proliferating upon treatment with specific MET inhibitors. We found that activation of HER family members in MET addicted cells, after MET inactivation, is able to increase cell viability *in vitro*, and to recover tumorigenicity *in vivo*. This observation is important if translated into a clinical context. In fact, gastric tumors that display *MET *gene amplification (10% of cases) are potentially addicted to MET expression and can be considered ideal targets for anti-MET therapies; however, aberrant activation of HER family members has also been shown to be concomitant in these tumors [36,37]. This means that the effect of MET inhibition could potentially be neutralized or attenuated by the parallel activation of receptors of the HER family. This implies that combinatorial inhibition of both MET and HER could likely improve the therapeutic effect. It is critical to underline that not all the growth factor-activated pathways can compensate for the lack of signal due to MET inhibition, as shown by data reported in this paper. Differently from previous observations in HER-addicted cells, the biological effects due to HER members activation was not due to their ability to trans-phosphorylate MET. In fact, the resistance was present not only in cells in which MET was inhibited by the specific small molecule, but also in cells in which the receptor was no longer present - and thus not available for trans-phosphorylation - due to shRNA-mediated silencing. These results suggest that the resistance induced by HER members activation may be rather due to their ability to activate signaling pathways that are critically overlapping with those generated by MET, such as activation of the AKT/MAPK pathways [20].

Finally, we have generated gastric cells resistant to a MET specific inhibitor and, upon ruling out the presence of *MET *gene amplification or mutations in either *MET *itself or other downstream signalling molecules such as RAS, Raf or PI3K (all of them being implicated in the acquisition of resistance to treatment with small molecules inhibitors), we found that the levels of HER2 and HER3 were significantly increased in these resistant cells. Moreover, HER3 silencing led to reversion of the resistance to MET inhibitors and to decreased cell viability. These data suggest that a molecular mechanism exploited by addicted cells to overcome the pro-apoptotic effect of MET inhibition may be the increased expression of HER family members, enhancing the sensibility to their cognate growth factors, which are usually available in the tumour microenvironment.

## Conclusions

In our work we studied the molecular mechanisms that could cause resistance to therapies targeting MET in gastric cancer. Altogether our data suggest that even in the cellular contexts that are more likely to respond to treatment with MET inhibitors, activation of HER family receptors -which is rather frequent in gastric tumors- can impair the biological response to treatment and can concur to the appearance of resistance. This should be taken in consideration in light of using new drugs or new association schemes that could concomitantly inhibit both these receptors and act synergistically.

## Methods

### Cell culture and compounds

SNU-5, NCI-H1993 cell lines were from ATCC, EBC-1 from JCRB. GTL16 cells were described in [15]. EGF was from Sigma (Milan, Italy), HRG1-β1 and IGF-1 from R&D; MSP was produced as in [38]. LY294002 was from Calbiochem, U0126 from Promega, PHA-665752 from Tocris Bioscience, Gefitinib from Sequoia Research Product. The EGFR L858R vector was kindly provided by Dr. Yarden [39]. TGFα was cloned in p156RRLsin.PPThCMV.MCS.pre [40]. The MET-shRNA was described in [10], the HER2-shRNA in [41]; the HER3-siRNA was from Sigma (MISSION siRNA, SASI_Hs01_00196190).

### Virus preparation, cell transduction and electoporation

Lentiviruses were produced as in [42]. Cells were transduced using 40 ng/ml of p24. Electroporation was performed with siRNAs 2 nM using the Cell line Nucleofector Kit V and Nucleofector II machine (Amaxa biosystems).

### Western blot analysis

Cells were starved in serum-free medium for 24 hours and then treated with EGF (5 ng/ml) or HRG1-β1 (10 ng/ml) for 10 minutes. Cells were lysed in LB buffer (2% SDS, 0.5 mol/L Tris-HCl (pH 6.8)). Primary antibodies: anti-phospho-Tyrosine (G10) (Upstate Biotechnology); anti-Actin (1-9), anti-HER3 (C-17) and anti-HER2 (C-18) (Santa Cruz Biotechnology); anti-vinculin (Sigma, Milan, Italy); anti-MET DL21 [43]; anti-phospho-MET Tyr1234/1235 (Cell Signaling Technology); anti-AKT, anti-phospho-AKT (Ser473), anti-p42/44 MAPK and anti-phospho-p42/44 MAPK Thr202/Thr204 (Cell Signalling Technology). Secondary antibodies were from Amersham. Detection was performed with ECL system (Amersham).

### Biological assays

Growth curves experiments were performed as in [25]; viability was evaluated on day.4, as in [44]. Growth in soft agar was performed as in [45], and was quantified with the Alamar Blue indicator dye (Trek Diagnostic Systems). Measurements were recorded using a DTX 880-Multimode plate reader (Beckman-Coulter).

### Real-Time PCR analysis

Total RNAs were extracted using TriReagent lysis buffer (Applied Biosystem). 2 μg of total RNA were retro-transcribed using random primers; cDNA was subjected to quantitative PCR, using Sybr green Master MIX (Applied Biosystem). Real-time PCR was performed with an ABI PRISM 7900HT (Applied Biosystems, Milan, Italy). Primer sequences are available from the authors.

### Xenograft transplantation experiments

3 × 10^5 ^tumor cells were injected subcutaneously in 6-week-old immunodeficient nu-/- female mice on a Swiss CD-1 background (Charles River Laboratories, Lecco, Italy). Tumor appearance was considered when tumor volume (calculated as in [46]) reached 100 mm^3 ^and monitored for 40 days. Animal procedures were approved by the Ethical Commission of the University of Turin and by the Italian Ministry of Health.

### Generation of cancer cell lines resistant to MET inhibitor

GTL16 cells were continuously cultured in the presence of a fix dose of PHA-665752 (500 nM), changing the media every 3 days. Resistance to MET inhibitor appeared in 6 months, after which cells were analyzed as described.

### Statistics

Results are means of at least three different independent experiments + standard error mean (s.e.m.) or standard deviation. Comparisons were made using the two-tailed Student's t-test.

## Competing interests

P.M. Comoglio received consultation fees from Bayer-Shering, Boehringer- Ingelheim, Johnson & Johnson and Servier. The other authors have no competing interests.

## Authors' contributions

SC and EG: study concept and design; execution of experiments; acquisition of data; analysis and interpretation of data; drafting of the manuscript. VC and JRS: design and execution of experiments (generation of resistant cell lines). CM: analysis and interpretation of data and statistical analysis. AB, LT and PMC: critical revision of the manuscript for important intellectual content. SG: study supervision and drafting of the manuscript. All authors read and approved the final manuscript.

## Supplementary Material

Additional file 1 **Expression levels of MET, EGFR, HER2 and HER3 in "MET addicted" gastric cancer cell lines**. The expression level of MET and EGFR family members was evaluated by Western blot and quantified by Geldoc (Quantity One program). The graph shows the relative expression of each receptor in GTL16, MKN45, SNU5 and Hs746T, normalized *versus *the expression level in 293 cells (non-tumoral cells). Click here for file

Additional file 2 **EGF and HRG1-β1 overcome MET inhibition in other MET-addicted cell lines**. A) Viability assay of MKN45, SNU5 and Hs746T gastric cancer cell lines. Cells were untreated (NT, *left columns*) or treated with MET inhibitor (PHA, 250 nM for MKN45 and Hs746T, 50 nM for SNU5, *right columns*) and stimulated or not with EGF (50 ng/ml) or HRG1-β1 (10 ng/ml). As shown, MET inhibition led to a strong decrease in cell viability compared to untreated cells, considered as 100%. Activation of HER family members, upon stimulation with EGF or HRG1-β1, conferred resistance to MET inhibition (*** P < 0,001). Hs746T cells couldn't respond to HRG1-β1 because they lack HER3 expression (data not shown). The specificity of the effect is shown by its loss in the presence of gefitinib (250 nM). B) Anchorage-independent growth assay, performed on MKN45 cells (SNU5 and Hs746T cell lines lack the ability to efficiently grow in soft agar). Cells were grown in agar for 2 weeks and the amount of viable cells forming colonies was quantified with the Alamar Blue dye. As shown, PHA-induced MET inhibition (250 nM) resulted in impairment of cell ability to grow in anchorage-independent manner. The stimulation with EGF (50 ng/ml) and - in a smaller extent - HRG1-β1 (10 ng/ml) rescued the ability of PHA-treated cells to form colonies. (* P < 0,05). The effect is abrogated in the presence of gefitinib (250 nM). Click here for file

Additional file 3 **The ability to overcome the effect of MET inhibition is not shared with other growth factors**. Viability assay of GTL16 cells untreated (NT, *left columns*) or treated with PHA (250 nM; PHA, *right columns*) and stimulated with different growth factors: EGF (50 ng/ml) *dark columns*, IGF (200 ng/ml) *light gray columns *and MSP (200 ng/ml) *dark gray columns*. As shown, only the treatment with EGF conferred resistance to MET inhibition. Click here for file

Additional file 4 **Silencing of HER2 and HER3 in wt and PHA-resistant GTL16 cells**. Western blot of total lysates of GTL16 cells (wt and resistant to PHA-GTL16 R500) probed with anti-HER2 (*left panel*), anti-HER3 (*right panel*) and anti-Vinculin. Bands were scanned and quantified. *Columns*, ratio between HER2 (*left panel*) or HER3 (*right panel*) and vinculin expression. Click here for file
