# Supplementary figures and images for: Activation of HER family members in gastric carcinoma cells mediates resistance to MET inhibition
Source: Mol Cancer. 2010 May 26;9:121. doi: 10.1186/1476-4598-9-121 (PMC2892452; doi:10.1186/1476-4598-9-121)

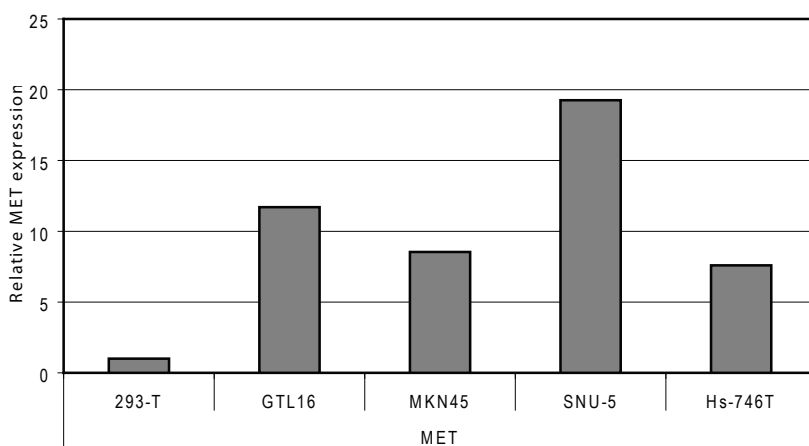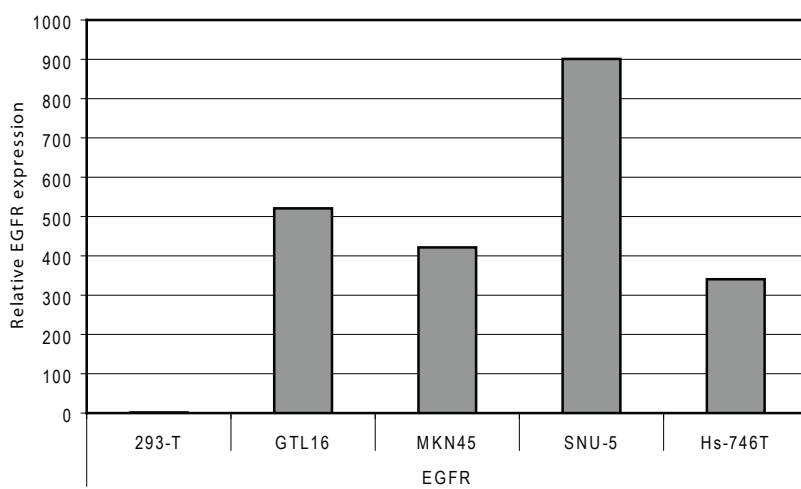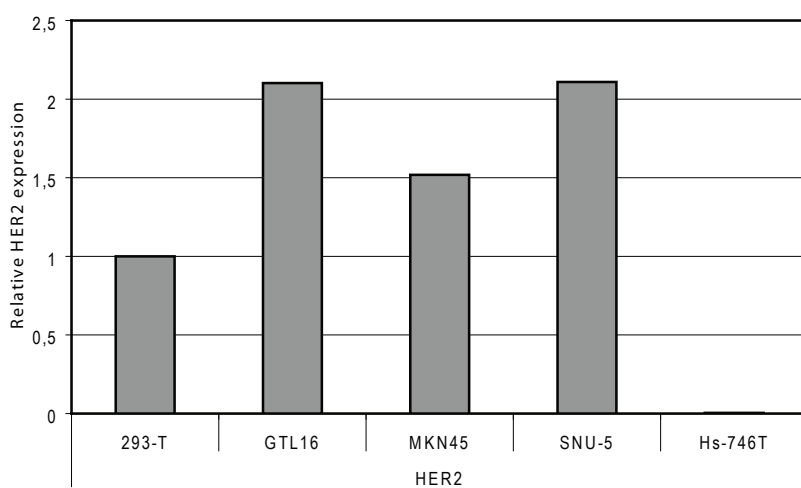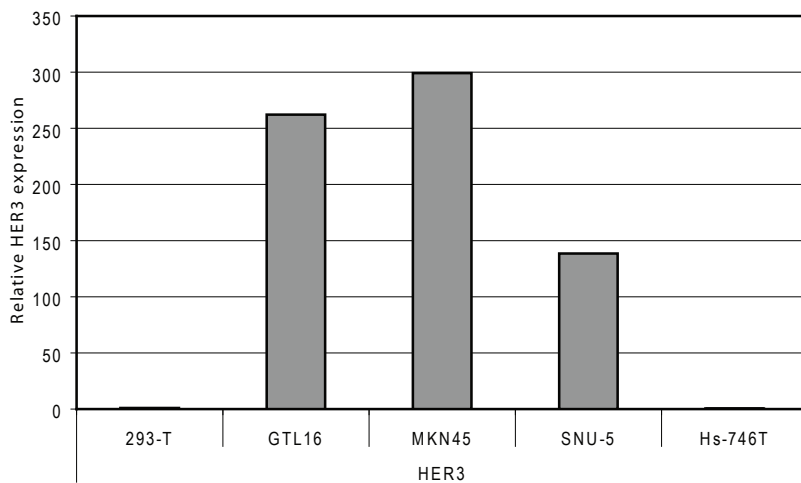

Supplement: Additional file 1 — Expression levels of MET, EGFR, HER2 and HER3 in "MET addicted" gastric cancer cell lines. The expression level of MET and EGFR family members was evaluated by Western blot and quantified by Geldoc (Quantity One program). The graph shows the relative expression of each receptor in GTL16, MKN45, SNU5 and Hs746T, normalized versus the expression level in 293 cells (non-tumoral cells). [file 1476-4598-9-121-S1.PDF]

A

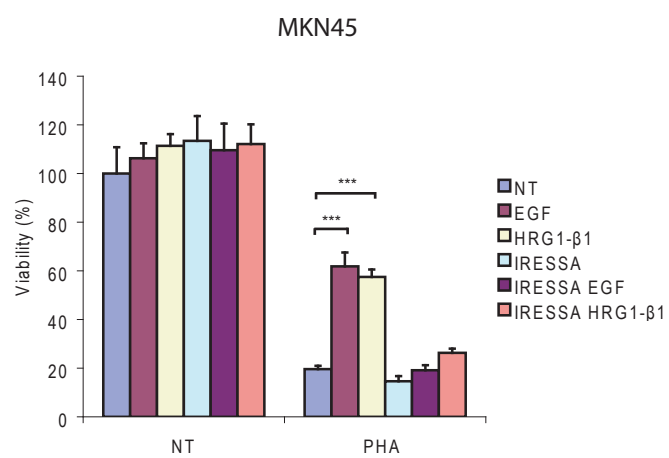

SNU-5

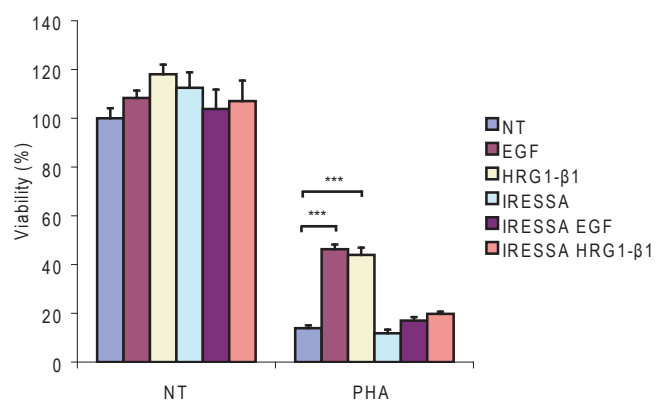

Hs746T

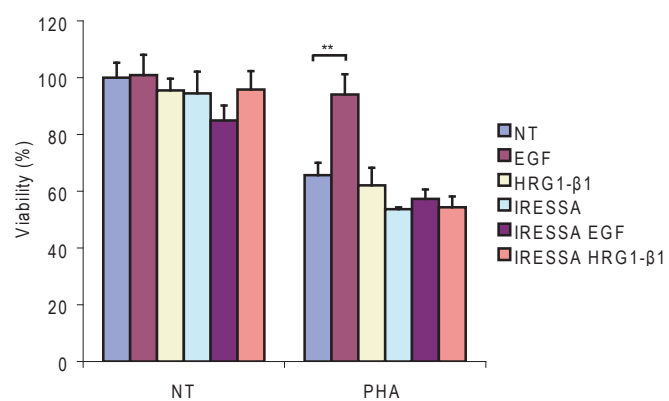

Viability assays

B

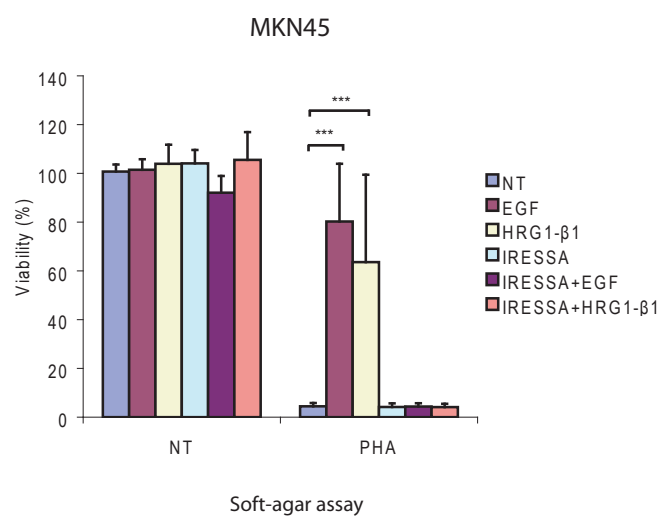

Supplement: Additional file 2 — EGF and HRG1-β1 overcome MET inhibition in other MET-addicted cell lines. A) Viability assay of MKN45, SNU5 and Hs746T gastric cancer cell lines. Cells were untreated (NT, left columns) or treated with MET inhibitor (PHA, 250 nM for MKN45 and Hs746T, 50 nM for SNU5, right columns) and stimulated or not with EGF (50 ng/ml) or HRG1-β1 (10 ng/ml). As shown, MET inhibition led to a strong decrease in cell viability compared to untreated cells, considered as 100%. Activation of HER family members, upon stimulation with EGF or HRG1-β1, conferred resistance to MET inhibition (*** P < 0,001). Hs746T cells couldn't respond to HRG1-β1 because they lack HER3 expression (data not shown). The specificity of the effect is shown by its loss in the presence of gefitinib (250 nM). B) Anchorage-independent growth assay, performed on MKN45 cells (SNU5 and Hs746T cell lines lack the ability to efficiently grow in soft agar). Cells were grown in agar for 2 weeks and the amount of viable cells forming colonies was quantified with the Alamar Blue dye. As shown, PHA-induced MET inhibition (250 nM) resulted in impairment of cell ability to grow in anchorage-independent manner. The stimulation with EGF (50 ng/ml) and - in a smaller extent - HRG1-β1 (10 ng/ml) rescued the ability of PHA-treated cells to form colonies. (* P < 0,05). The effect is abrogated in the presence of gefitinib (250 nM). [file 1476-4598-9-121-S2.PDF]

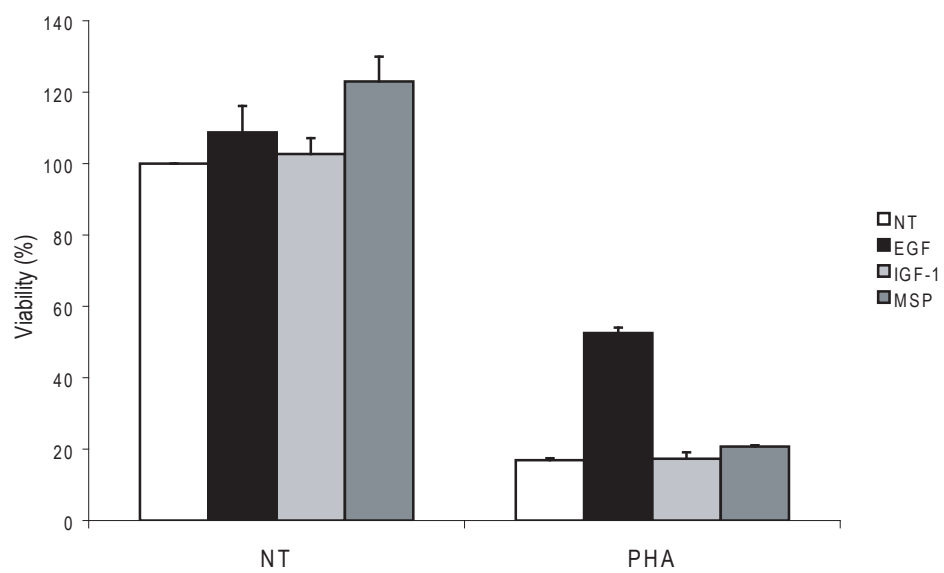

Supplement: Additional file 3 — The ability to overcome the effect of MET inhibition is not shared with other growth factors. Viability assay of GTL16 cells untreated (NT, left columns) or treated with PHA (250 nM; PHA, right columns) and stimulated with different growth factors: EGF (50 ng/ml) dark columns, IGF (200 ng/ml) light gray columns and MSP (200 ng/ml) dark gray columns. As shown, only the treatment with EGF conferred resistance to MET inhibition. [file 1476-4598-9-121-S3.PDF]

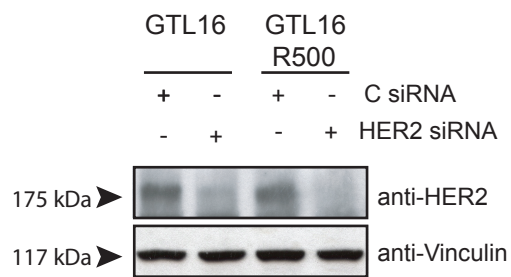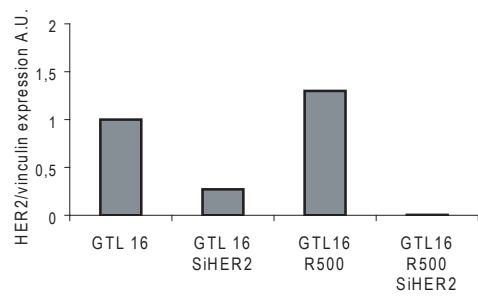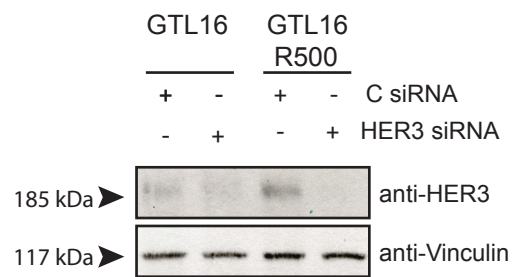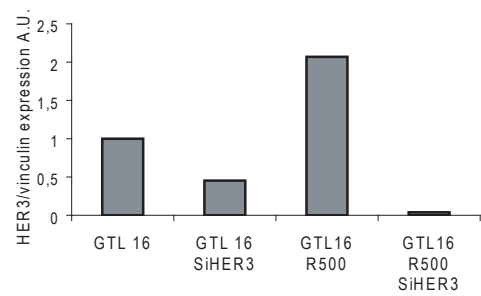

Supplement: Additional file 4 — Silencing of HER2 and HER3 in wt and PHA-resistant GTL16 cells. Western blot of total lysates of GTL16 cells (wt and resistant to PHA-GTL16 R500) probed with anti-HER2 (left panel), anti-HER3 (right panel) and anti-Vinculin. Bands were scanned and quantified. Columns, ratio between HER2 (left panel) or HER3 (right panel) and vinculin expression. [file 1476-4598-9-121-S4.PDF]
